# Limitations on Monaural and Binaural Temporal Processing in Bilateral Cochlear Implant Listeners

**DOI:** 10.1007/s10162-015-0527-7

**Published:** 2015-06-24

**Authors:** Antje Ihlefeld, Robert P. Carlyon, Alan Kan, Tyler H. Churchill, Ruth Y. Litovsky

**Affiliations:** Department of Biomedical Engineering, New Jersey Institute of Technology, 645 Fenster Hall, 323 Martin Luther King Blvd, Newark, NJ 07102 USA; Waisman Center, 1500 Highland Avenue, Madison, WI 53705 USA; MRC Cognition and Brain Sciences Unit, 15 Chaucer Rd, Cambridge, CB2 7EF UK

**Keywords:** cochlear implant, rate sensitivity, interaural time difference, pitch

## Abstract

Monaural rate discrimination and binaural interaural time difference (ITD) discrimination were studied as functions of pulse rate in a group of bilaterally implanted cochlear implant users. Stimuli for the rate discrimination task were pulse trains presented to one electrode, which could be in the apical, middle, or basal part of the array, and in either the left or the right ear. In each two-interval trial, the standard stimulus had a rate of 100, 200, 300, or 500 pulses per second and the signal stimulus had a rate 35 % higher. ITD discrimination between pitch-matched electrode pairs was measured for the same standard rates as in the rate discrimination task and with an ITD of +/− 500 μs. Sensitivity (*d*′) on both tasks decreased with increasing rate, as has been reported previously. This study tested the hypothesis that deterioration in performance at high rates occurs for the two tasks due to a common neural basis, specific to the stimulation of each electrode. Results show that ITD scores for different pairs of electrodes correlated with the lower rate discrimination scores for those two electrodes. Statistical analysis, which partialed out overall differences between listeners, electrodes, and rates, supports the hypothesis that monaural and binaural temporal processing limitations are at least partly due to a common mechanism.

## INTRODUCTION

Cochlear implants (CIs) can restore hearing to profoundly deaf individuals. The most successful CI listeners have very good speech intelligibility in quiet listening conditions, but this intelligibility is much reduced in the presence of interfering sound. In those acoustically crowded settings, normally hearing listeners can use differences between the pitches and spatial locations of competing sources as potent cues for separating sound, thereby allowing the listener to attend to the source of interest (Darwin and Hukin 2000; Brungart and Simpson 2002; Freyman et al. 2005; Kidd et al. 2005; Rakerd et al. 2006; Ihlefeld and Shinn-Cunningham 2008; Maddox and Shinn-Cunningham 2012).

CIs are capable of conveying cues to pitch both through place of activation and through temporal patterns of stimulation (McKay et al. 2000; Donaldson et al. 2005; Carlyon et al. 2010a). In the case of bilateral implantation, CI listeners can also access information for spatial source location through interaural level differences (ILDs) and interaural time differences (ITDs; van Hoesel and Tyler 2003; Seeber and Fastl 2008).

For typical configurations of listener head size and room reverberation, the first major spatial cue, ILDs reaching a listener’s ears, can encode sound location at high frequencies (Hartmann 1983; Rakerd and Hartmann 2004; Ihlefeld and Shinn-Cunningham 2011). In everyday clinical use, however, the two CI processors adjust gain control independently across ears, severely limiting the fidelity of ILDs (e.g., Laback et al. 2004, 2015). The second major spatial cue is conveyed through ITDs. Clinical CI processors often rely on stimulation rates of 900 pulses per second (pps) or higher, although lower rates are sometimes used, and the timing of the pulses is neither synchronized between the two processors nor to the input stimuli at the two ears. As a result, only envelope ITD cues are accessible to many CI listeners. When only ILD and envelope ITD cues are available, this can severely limit the benefit of spatial cues to CI listeners (van Hoesel and Tyler 2003). Provision of fine-structure ITD information may improve a CI listener’s ability to utilize spatial cues when listening for a target in the presence of background sounds (Ihlefeld and Litovsky 2012) but only if listeners can effectively process that information. However, experimental processing strategies that explicitly provide fine-structure ITD cues have not succeeded in improving either sound segregation or localization, suggesting that the ability of CI users to exploit fine timing cues is limited, even when that information is explicitly encoded (van Hoesel et al. 2008; Loizou et al. 2009). Recent work shows that a split-rate strategy, with slow-rate fine-structure ITD cues at the four apical electrodes and high-rate envelope cues at the basal electrodes, can preserve good lateralization and speech perception in quiet. However, performance is still poorer than that seen in normally hearing listeners (Churchill et al. 2014).

Degraded auditory processing of fine timing cues for CI listeners, compared to those with normal hearing, has been reported not only for the binaural ITD discrimination task but also for a monaural task involving rate discrimination. A severe limitation of both unilateral and bilateral CIs is that sensitivity to temporal information deteriorates dramatically at pulse repetition rates faster than about 300 pps (Shannon 1983; Townshend et al. 1987; Kong et al. 2009; van Hoesel 2008; van Hoesel et al. 2009; Venter and Hanekom 2014). This “high-rate limitation” has been observed both in rate discrimination tasks with unilaterally implanted CI listeners (Shannon 1983; Townshend et al. 1987; Zeng 2002; Baumann and Nobbe 2004; Kong et al. 2009; Bahmer and Baumann 2013) and for the detection of ITDs by bilaterally implanted CI listeners (van Hoesel et al. 2002; van Hoesel and Tyler 2003; Laback et al. 2007; van Hoesel 2007; van Hoesel et al. 2009). For the majority of CI listeners, rate discrimination becomes very poor or impossible for rates above 300 pps, although marked individual differences have been observed. Similarly, ITD thresholds typically increase with increasing pulse rate, and for rates above 300 pps, CI listeners tend to rely more heavily on onset ITD as opposed to ITD in the ongoing portion of sound (van Hoesel 2008). Even for listeners who have retained good binaural sensitivity, perhaps because they have had late-onset deafness (Litovsky et al. 2010), ITD sensitivity generally declines for rates above 600 pps (Laback and Majdak 2008; van Hoesel et al. 2009).

The neural basis for the high-rate limitations in unilateral rate and bilateral ITD processing remains unknown. The question of whether sensitivities to monaural rate and binaural timing cues are limited by a shared auditory mechanism is unresolved and is the focus of the current study.

One possibility is that, in both cases, the limitation arises at the level of the auditory nerve. Some studies report that auditory nerve fibers can follow electrical pulse trains even at rates above 1000 pps (Dynes and Delgutte 1992; Litvak et al. 2001). There is, however, evidence that, at rates above 400 pps, the fidelity of auditory nerve phase locking is degraded in both acutely and long-term deafened cats, when compared to normally hearing controls (Shepherd and Javel 1997).

It is also possible that high-rate limitations arise more centrally. Most CI users have experienced a prolonged period of deafness prior to implantation, and it is known that in addition to causing auditory nerve degeneration, hearing deprivation can strongly alter membrane and synaptic properties in the central nervous system by decreasing the amount of inhibition expressed along nearly all nuclei in the auditory system (Takesian et al. 2009). Moreover, previous work shows that at the level of the inferior colliculus, hearing deprivation affects the binaural response properties to electrically delivered ITD by reducing the number of ITD-sensitive neurons in congenitally as compared to acutely deafened animals and by altering the ITD tuning curves in the neurons that remain ITD sensitive (Hancock et al. 2010). Further evidence for central processing limitations stems from recent work, where for the same human listeners and stimuli, amplitude fluctuations in the electrically evoked compound action potentials to single-electrode monaural pulse trains were compared to behavioral rate discrimination performance. For high-rate pulse train stimuli, the amplitude fluctuations present in the evoked compound action potential were not sufficient to account for the breakdown in rate discrimination (Carlyon and Deeks 2013).

The present study examined the high-rate limitations in monaural rate sensitivity and binaural ITD sensitivity by measuring performance on both tasks in the same bilaterally implanted CI listeners. The aim was to determine whether the two measured limitations share a common mechanism. Because the primary interest was in the deterioration in performance at high rates, here, stimuli were designed such that there was a sufficiently large difference between standard and signal stimuli, so that, at low rates, discrimination should be easy. ITD discrimination was measured for three pitch-matched electrode pairs, located at the apical, middle, and basal regions of the electrode array and for pulse rates of 100, 200, 300, and 500 pps. Monaural rate discrimination was measured at the same baseline rates, separately for the same three electrodes in each ear (i.e., a total of six electrodes).

Across-electrode differences in ITD performance at high rates could be primarily caused by deficits specific to the binaural system or could reflect across-electrode differences in temporal encoding that also affect monaural tasks. If variations in ITD discrimination were primarily driven by a deficit in the input to the binaural system that is common to binaural and monaural tasks, then there should exist a correlation between ITD discrimination of each electrode pair and the poorer of the rate discrimination scores in the two electrodes of that pair. When evaluating this hypothesis, it is important to rule out factors that should covary across the two tasks. Because there is existing evidence that performance in both tasks is worse at high rates, rate was removed as a factor when calculating correlations. Similarly, because there are multiple factors that could cause performance to covary across listeners for all conditions, ranging from neural survival to cognitive capacity, the correlation excluded effects of overall differences in performance across listeners and across sites of stimulation.

The specific hypothesis was that ITD discrimination and “worse ear” rate discrimination should correlate when those overall factors of electrode, rate and listener were statistically partialed out and that the correlation should be observed in an electrode-specific manner. For example, ITD discrimination on an apical electrode pair should be predictable from the worse rate discrimination performance from the two constituent apical electrodes, but not from the middle or basal electrodes of that listener.

## METHODS

Eight bilateral CI listeners, all postlingually deafened and implanted with Nucleus devices (Cochlear®), were tested and paid for their time. All testings were administered according to the guidelines of the Institutional Review Board of the University of Wisconsin-Madison. Table 1 lists details of each listener’s etiology.

**TABLE 1:** Clinical etiology, device: Nucleus 24 electrodes

CI listener	IAJ	IAZ	IBD	IBK	IBM	IBP	IBR	ICE
Device	CI24M Left; CI24R (CS) Right	Freedom (CA) Left; Freedom (CA) Right	CI24M Left; CI24M Right	CI24R (CS) Left; Freedom (CA) Right	CI512 Left; Freedom (CA) Right	CI24M Left; CI24M Right	CI24R (CS) Left; CI512 Right	Freedom (CA) Left; Freedom (CA) Right
Strategy	ACE	ACE	SPEAK	ACE	ACE	ACE	ACE	ACE
Etiology	Unknown	Unknown	Meniere’s/Noise/Hereditary	Hereditary	Unknown	Hereditary	Progressive sensorineural	Unknown
Hearing loss onset age (years)	5	55	36	53	30	55	28	66
Age (years)	65	77	88	57	57	70	56	73
Use duration (months)	R (84)	R (49)	R (244)	R (48)	R (10)	R (25)	R (7)	R (53)
	L(168)	L (72)	L (244)	L (48)	L (55)	L (96)	L (4)	L (47)

## STIMULI

### Overall Design

Stimulus presentation and response recording were similar to our previous methods (Ihlefeld et al. 2014). Specifically, we implemented custom-written experimental software in Matlab (The MathWorks, Natick, MA). Two synchronized L34 research processors (Cochlear Ltd., Sydney, NSW, Australia) delivered the stimuli to the listeners’ internal devices. Oscilloscope readings of the outputs from two test implants verified proper function of the custom-written stimulation software. Moreover, at the beginning of each testing day, oscilloscope readings confirmed proper function of the testing equipment. Electrodes were activated in monopolar configuration (MP1 + 2). Pulses were biphasic, with the initial phase cathodic, 45 μs phase duration and 8 μs phase gap.

### Electrode Selection

#### 25-pps Loudness Rating

At the beginning of each experiment, listeners performed a loudness rating task for all even electrodes in both ears, with constant-amplitude, 25-pps, 500-ms pulse trains, in order to measure the most comfortable level (MCL). Electrodes that were deactivated in the listeners’ everyday maps were excluded from testing. Interpolation of MCL on all even electrodes yielded MCL for all odd electrodes, resulting in a map of MCL at 25 pps for all 44 electrodes (in both ears).

#### Direct Pitch Comparison

Listeners performed an across-ear place pitch matching task with 25-pps pulse trains (Carlyon et al. 2010b; Macherey et al. 2011; Macherey and Carlyon 2012). The aim of this task was to choose “matched” pairs that, as closely as possible, produced the same place of excitation in the two ears. The 25-pps rate was used because, at this rate, listeners’ pitch judgments depend on place-of-excitation cues, and the temporal cues correspond to a rate below the lower limit of pitch. Specifically, the upper limit of temporal pitch can vary across electrodes and, presumably, across ears. Therefore, in a given listener, pitch matching with higher-rate pulse trains can lead to biased or unreliable measures of place pitch (Macherey et al. 2011; Macherey and Carlyon 2012). To obtain pitch-matched estimates, a 25-pps pulse train at MCL was presented to the left-ear electrode, followed by 500-ms silence, and then by a 25-pps pulse train at MCL presented to the right ear. In a two-interval, two-alternative forced choice (2I-2AFC) task, the listener reported whether the sound in the second interval was lower or higher in pitch than the sound in the first interval. No feedback was provided.

The experimenter selected three left-ear electrodes (basal/mid/apical) at or adjacent to electrodes L4, L12, L20. For each of the three left-ear electrodes, a bracket of five right-ear electrodes was tested that surrounded each of the three left-ear electrodes. In order to control for perceptual range biases (Macherey and Carlyon 2012), the selection of right-ear electrodes varied from block to block, alternating the center of the right-ear electrode bracket. For instance, a lower-bracket block for electrode L4 could consist of the pairs [L4-R1, L4-R2, L4-R3, L4-R4, L4-R5], whereas a higher-bracket block could consist of the pairs [L4-R3, L4-R4, L4-R5, L4-R6, L4-R7]. All left/right pairs were interleaved randomly from trial to trial and presented five times per block. Therefore, each block contained 75 trials (3 × 5 × 5) and lasted about 5 to 10 min.

Six blocks (i.e., 30 trials per electrode pair) were collected. Probit line fits to lower-bracket and to higher-bracket block performance curves established for each bracket the three electrode pairs closest to 50 %; i.e., the point of subjective equality. If the point of subjective equality differed by more than one electrode between lower- and higher-bracket blocks, the measurement was repeated; otherwise, new probit fits and corresponding points of subjective equality were derived by combining lower- and higher-bracket raw data. From these final probit fits, three bilaterally pitch-matched electrode pairs were identified: a basal, an apical, and a midrange pair. Only those six electrodes were tested for the remainder of the experiment.

### Loudness Calibrations

#### Standard and Signal Rate Loudness Rating

The main experiment measured ITD discrimination at 100, 200, 300, and 500 pps, and rate discrimination between these standard rates and corresponding signal rates were 35 % higher than the standard rates. Threshold levels (T-levels) and MCLs were mapped for each of these eight rates at each of the six pitch-matched electrodes [6 electrodes × 8 rates × 2 (T/C level) = 96 ratings] for five listeners. Due to an oversight, for two listeners, ICE and IBD, the T-levels were treated as zero throughout the experiment. For all subsequent tasks, the total duration of each stimulus remained 500 ms, and stimuli were gradually ramped on and off, with 100 ms long onset and offset Hanning-shaped ramps. Ramps were applied from T-level to the desired amplitude, in order to reduce the possibility of listeners performing the ITD task based on the first pulse in each pulse train (van Hoesel 2007). This was deemed important because the current study examines the relationship between performance in ITD and rate discrimination tasks and because only the ITD task could potentially be performed using only the first pulse. Note, however, as shown in the “Results” section, performance levels for IBD and ICE, where ramps started at zero, were not better than for the other listeners.

#### Standard Rate Loudness Balancing

Each listener performed 21 loudness comparisons in a two-interval loudness balancing task. First, for each of the three electrode pairs, a 100-pps pulse train was played to the left and right ear sequentially, and the listener adjusted the right-ear stimulus until both ears sounded equally loud, following a method described by Landsberger and McKay (2005). The right-ear stimulus was initially much softer than its MCL, then the listener increased its level until it was louder than the left-ear sound, then decreased it again until it was softer than the left-ear sound, and then finally increased the level until both sounds were equally loud. The same procedure was repeated with the right-ear stimulus fixed, and the left-ear stimulus to be adjusted. The level difference outcomes were then averaged across the two tracks, resulting in an updated MCL for the left ear.

Listeners then performed unilateral loudness balancing, calibrating within each ear and electrode 100 versus 200 pps, 100 versus 300 pps, and 100 versus 500 pps [6 electrodes × 3 comparisons = 18 ratings]. From the resulting updated map, at MCLs, all standard rates should sound equally loud across electrodes and ears.

#### ILD Centering

Listeners performed a one-interval ILD centering task with a zero ITD. For each electrode pair and rate, a pulse train was played to the left and right ear simultaneously. If listeners did not perceive the sound in the center of the head, they could turn down the level of the pulse train in the ear where the sound was perceived by a small amount of not more than 5 current units [3 electrode pairs × 4 standard rates = 12 ratings], where one current unit corresponds to a difference of approximately 0.17 dB. The outcome of the ILD centering was a newly updated level map. At the standard rate, each stimulus sounded approximately centered when played simultaneously to both ears, while still sounding approximately equally loud when played sequentially across ears. These new levels were used both in the ITD task and for the standard rates used in the rate discrimination task.

#### Same Ear, Same Electrode: Standard Rate Versus Signal Rate Loudness Balancing

The signal levels for the rate discrimination tasks were matched in loudness to their corresponding standard level, as determined from the procedures described in the last three subsections. Specifically, listeners performed another series of two-interval loudness balancing tracks, calibrating for each electrode standard versus signal rates (i.e., 100 vs. 135 pps; 200 vs. 270 pps; 300 vs. 405 pps; 500 vs. 675 pps for each of the 6 electrodes, 24 ratings). The resulting final level map also ensured that standard and signal rates sounded equally loud within each ear.

## ITD AND RATE DISCRIMINATION

### ITD Task

Listeners performed a 2I-2AFC ITD discrimination task using the method of constant stimuli. Only standard rates were tested. Stimuli in each interval were either a left-leading pulse train followed by right-leading, or vice versa. Stimuli were 500 ms long, the interstimulus interval was 500 ms, and the ITD was fixed at 500 μs. The listener’s task was to indicate whether the sound moved from left to right across the two intervals or from right to left. Correct-answer feedback was provided.

Within each block, the stimulus pulse rate varied randomly among the standard rates from trial to trial such that each standard rate was presented once before all standard rates were repeated. The electrode pair was fixed within a block and varied in a Latin square balanced design across blocks. Most listeners performed a total of 12 blocks of 64 trials each, resulting in 64 trials per electrode pair and standard rate. Depending on the availability of each listener, and on their overall response speed, a few listeners completed fewer trials in one or more conditions. Specifically, listener IBR completed 16 trials at the apical electrodes, and listener IBM completed 32 trials at each of the electrode pairs.

### Rate Task

For each of the six electrodes, listeners performed a 2I-2AFC rate discrimination task. Stimuli either consisted of the standard rate in the first interval and the signal rate in the second interval or vice versa. Signal rates were 35 % higher than the standard rates. The listener’s task was to report which interval contained the higher pitch. Correct-answer feedback was provided.

Within each block, we varied the stimulus pulse rate randomly among standard rates from trial to trial, such that each standard rate was presented once before all of them were repeated. The electrode was fixed within a block and varied in a Latin square balanced design across blocks. Listeners performed a total of 24 blocks of 64 trials each, resulting in 64 trials per electrode, ear, and standard rate.

## STATISTICAL ANALYSIS

Percent correct scores were converted to *d*′ scores, averaging the z-scores of the correct responses across the two response intervals, to correct for bias (Klein 2001). Data were analyzed with repeated measures analysis of variance (ANOVA) using SPSS version 22 (IBM-SPSS Inc., Somers, NY, USA). To test the hypothesis that the performance deterioration for the rate discrimination and ITD tasks at high pulse rates arises from common processing limitations, we conducted a correlation analysis to assess how closely monaural rate discrimination in the worse ear could predict ITD sensitivity. ITD scores were entered as the dependent variable into a univariate ANOVA. Listener, electrode, and rate were treated as fixed factors to partial out their effects on ITD variance (Bland and Altman 1995). The ANOVA modeled the main effects of, but not the interactions between, these fixed factors. Any remaining effect of worse-ear rate discrimination on ITD performance scores would therefore reflect the covariation between worse-ear rate discrimination and ITD discrimination, while modeling and controlling for any main effects of listener, rate, or electrode position.

For experimental designs with repeated measurements, the method by Bland and Altman (1995) calculates correlation coefficients within listeners, computationally equivalent to calculating the correlation coefficient between normalized performance scores. Specifically, separately each of the two tasks *T*, worse-ear rate and ITD discrimination, all *d*′ scores for a given listener *L* are averaged across all rate *Rt* and electrode *El* conditions for that task, and the resulting within-listener grand means are subtracted from every performance score for that listener:

1 $$ \overline{d}{\prime}_{L,\  El,\ Rt,\ T} = d{\prime}_{L,\  El,\ Rt,\ T}-\frac{1}{N_{Rt}{N}_{El}}{\displaystyle {\sum}_{r=1}^{Rt}{\displaystyle {\sum}_{e=1}^{El}d{\prime}_{L,\  El,\ Rt,\ T}}} $$

where *N*_*Rt*_ and *N*_*El*_ are the number of tested rates and electrodes, respectively.

For each rate and task, the mean of $\overline{d}{\prime}_{L, El,Rt,\ T}$ across listeners and electrodes is calculated:

2 $$ \overline{d}{\prime}_{Rt,T} = \frac{1}{N_L{N}_{El}}{\displaystyle {\sum}_{l=1}^L{\displaystyle {\sum}_{e=1}^E\overline{d}{\prime}_{L, El,Rt,T}}} $$

where *N*_*L*_ equals the number of listeners.

For each electrode and task, $\overline{d}{\prime}_{Rt,T}$ is subtracted from the corresponding $\overline{d}{\prime}_{L,\  El,\ Rt,\ T}$:

3 $$ d{\prime}_{El,\ T}=\overline{d}{\prime}_{L, El,Rt,T} - \overline{d}{\prime}_{Rt,T} $$

For each of the three electrodes and both tasks, this difference is averaged across all rates and listeners:

4 $$ \overline{d}{\prime}_{El,T}=\frac{1}{N_L{N}_{Rt}}{\displaystyle {\sum}_{l=1}^L{\displaystyle {\sum}_{r=1}^Rd{\prime}_{El,T}}} $$

Finally, for both tasks, normalized performance scores *d* ′ _norm *L*,*El*,*Rt*,*T*_ are defined as the difference:

5 $$ d{\prime}_{\mathrm{norm}\ L, El,Rt,T}=d{\prime}_{El,T}-\overline{d}{\prime}_{El,T} $$

An alternative and widely used method that could be applied to the rate and ITD discrimination scores measured here is the linear mixed effects model (Xu 2003). The approach compares two mixed models, one with only dummy variables (for the current study, listener, rate, and electrode position) and another model with those dummy variables plus the fixed effect of interest (for the current study, worse-ear rate discrimination). It is less conservative than Bland and Altman’s method, because it ignores any variability that cannot be modeled by any of the factors. The current results are primarily discussed in terms of Bland and Altman’s method, because of its equivalence to the normalized correlation. However, for completeness, the results obtained with the linear mixed effects model are also reported.

## RESULTS

### Loudness Balancing

With the exception of listener IAZ, all listeners adequately performed the initial loudness balancing task. A loudness balancing track was considered adequate when a listener completed at least two loudness reversals (i.e., making the tracked signal stimulus first louder, then softer, as compared to the standard stimulus). Except for IAZ, all listeners completed two or more reversals per tracking. However, over a wide range of signal and standard rates and levels, IAZ never completed more than one reversal and often performed no reversal. Indeed, IAZ reported perceived differences between standard and signal stimuli but could not rate consistently which of the two sources was softer and which was louder. The data from listener IAZ were therefore excluded from further analyses and discussion.

### ITD Task

**FIG. 1:**
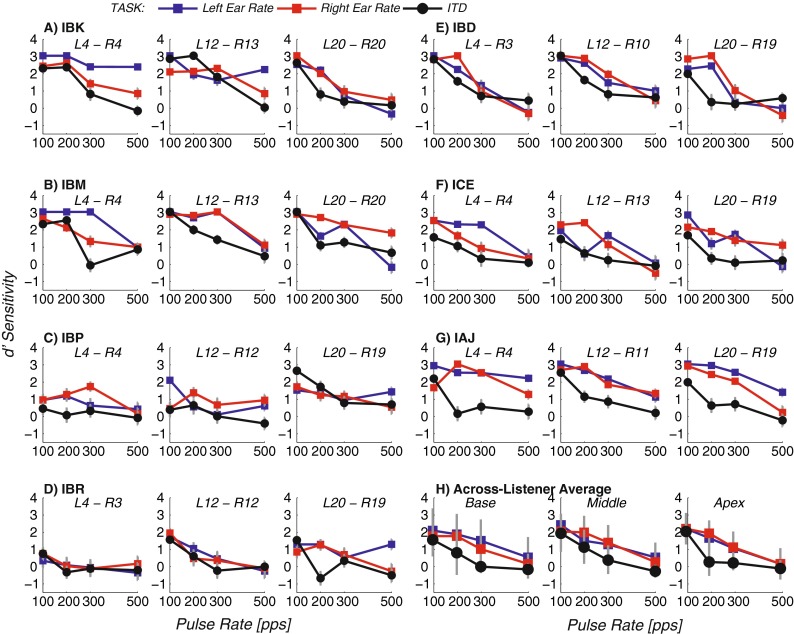
Rate and ITD sensitivity as a function of pulse rate. Panels **A**–**G** show results for one CI listener and stimulation site, and panel **H** shows the average results across listeners.

The black lines with circles in each panel of Figure 1 show performance in the ITD task. Each panel shows *d*′ scores for one electrode pair and CI listener; note that higher electrode numbers correspond to more apical stimulation. Figure 1A–G shows the results for each individual listener, and Figure 1H shows the average performance across listeners. Error bars show estimates of the 95 % confidence intervals. For Figure 1A–G, confidence intervals were calculated as 1.96 times the standard error across trials for each listener, electrode, and task on the assumption of a binomial response distribution, for Figure 1H, confidence intervals were calculated as 2.45 times the standard error of the mean across listeners (*t*_0.975, 6_ = 2.45).

A repeated measures analysis of variance (ANOVA) revealed the strong effect of baseline rate apparent in Figure 1 [*F*_(3,18)_ *=* 39.68, *p <* 0.001]. There was no significant effect of electrode location [*F*_(2,12)_ *=* 0.67, *p =* 0.53].

Performance deteriorated with increasing rate in the individual data for nearly all listeners and electrode pairs. The only exceptions were the basal and middle pairs for listener IBP, where discriminability was poor at all rates tested. Although there was no overall effect of electrode position in the mean data, in some cases, an individual listener’s scores differed across electrodes. For example, listener IBP performed better on the apical than on the middle pair, whereas the opposite was true for listener IBK.

### Rate Task

The red and blue lines with squares in Figure 1 show rate discrimination performance for the right and left ears, respectively. With increasing rate, *d*′ decreased without any obvious overall effect of ear or electrode position. These trends were confirmed by a repeated measures ANOVA, which revealed a significant effect of rate [*F*_(3,18)_ = 24.17, *p <* 0.001], but no significant effects of ear or of electrode location [*F*_(1,6)_ *=* 1.39, *p =* 0.28; *F*_(2,12)_ *=* 0.20, *p =* 0.82, for ear and electrode location, respectively]. Although there was no overall effect of ear of presentation in the mean data, rate discrimination did sometimes differ markedly between the two ears, most notably at the 500-pps rate for listeners IBK (basal pair), IBM (apical pair), and IAJ (apical pair). Similarly, although rate discrimination performance was generally similar across the three electrode positions, there were instances where it was better either for the basal (e.g., listener IBK, left ear) or apical (IBM, right ear) site.

### Correlations Between ITD and Rate Discrimination

**FIG. 2:**
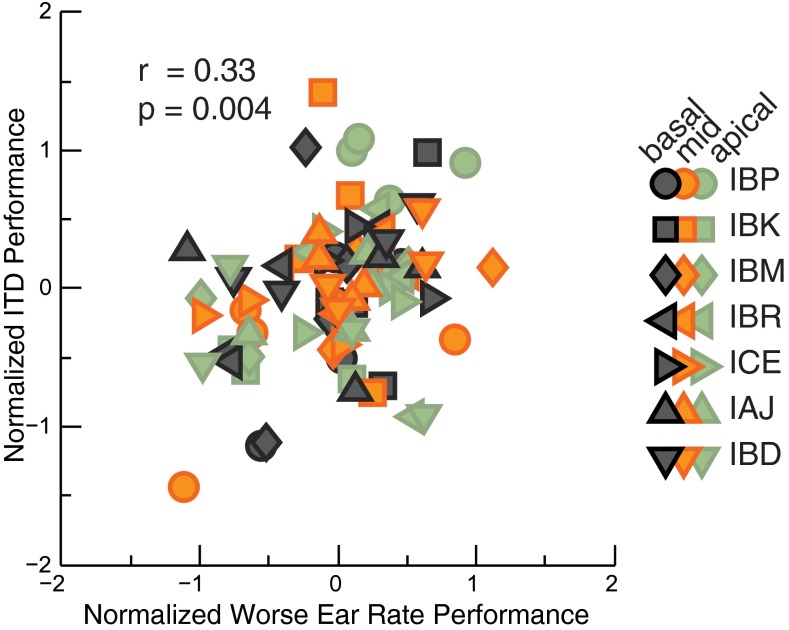
Normalized *d*′_norm_ sensitivity scores for worse-ear rate performance versus ITD (cf. Eq. 4). Different listeners are shown by *different symbols*, and *colors* denote the place of stimulation. *Repeated symbols* reflect the fact that performance is shown across a range of pulse rates.

Figure 2 depicts normalized performance scores, *d* ′ _*norm L*,*El*,*Rt*,*T*_, for monaural rate discrimination in the worse ear (abscissa) versus ITD sensitivity (ordinate). Each symbol denotes performance by an individual listener; the three colors grey, orange, and green represent basal, mid, and apical sites of stimulation, respectively. Results show that worse-ear rate performance did indeed account for a significant additional amount of variability in the ITD performance. The residual correlation was 0.33 [*F*_(1,71)_ = 8.79, *p =* 0.004], corresponding to worse-ear rate discrimination accounting for an additional 11 % of the variance. The correlation was larger than that obtained from better-ear rate sensitivity [1 % of variance, not significant, *F*_(1,71)_ = 0.94, *p* = 0.34], or from across-ear average rate scores [6 % of variance, *F*_(1,71)_ = 1.377, *p* = 0.03].

The 11 % of variance in the ITD scores accounted for by worse-ear rate discrimination is a conservative estimate, as this percentage is in addition to the main effects of rate [39 % of variance, *F*_(3,71)_ *=* 15.11, *p <* 0.001], listener [24 % of variance, *F*_(6,71)_ *=* 3.79, *p* = 0.002], and electrode [2 % of variance, not significant, *F*_(2,71)_ *=* 1.08, *p =* 0.34] on ITD performance. It is possible that those main effects are also influenced by the same common factor that accounts for the additional 11 % of worse-ear rate and ITD correlation. It is also worth noting that the less conservative linear mixed effects model approach (Xu 2003) led to a larger estimate—27.9 %—of the variance in ITD scores accounted for by worse-ear rate discrimination.

Three additional checks support the interpretation that the worse of the two rate performance scores in both ears can account for a significant proportion of the variance in the ITD performance: First, because performance was often at ceiling at the two lower rates, the use of listener as a fixed factor may not have completely removed the effects of between-listener differences in sensitivity. That is, some listeners might have had greater sensitivity at all rates, but this may only have been apparent at the higher rates where performance was below ceiling. With those two lower rates removed, the analysis was therefore repeated. The amount of variance accounted for increased slightly to 16 %, and the corresponding correlation of 0.4 was significant [*F*_(1,31)_ = 5.96, *p =* 0.02]. Second, it was of interest to check whether worse-ear rate performance was predictive of ITD performance only at place-matched electrode positions. When labeling the three electrode positions “a” for apical, “m” for mid, and ”b” for basal, then the original place-matched analysis used the following pairings of ITD and worse-ear rate performance: [(a,a); (m,m); (b,b)]. We performed two additional correlation analyses, with ITDs and worse-ear rate performance “shuffled” scores across electrodes. One shuffled analysis used pairings [(b,m); (m,a); (a,b)], whereas the other used pairings [(b,a); (m,b); (a,m)]. In both cases, the amount of variance explained was less than 5 % [*F*_(1,71)_ = 0.5, *p* = 0.482 for first shuffling, *F*_(1,71)_ = 0.338, *p* = 0.563 for second shuffling], and the correlation was not significantly different from zero and was significantly smaller than in the unshuffled case (one-tailed *t* test, first shuffling: *t*_(70)_ = 2.07, *p* < 0.05, second shuffling *t*_(70)_ = 1.71, *p* < 0.05).

Because the rate and ITD discrimination measurements were obtained at the same level for each condition, a third test checked whether stimulus level could account for the covariation in the scores for the two tasks. To that end, stimulus level was entered as an additional covariate into the original (unshuffled) analysis. The effect of worse-ear rate discrimination remained significant [*r =* 0.32, *F*_*(*1,70)_ *= 7.84, p <* 0.01], whereas stimulus level did not account for a significant proportion of the variance [*r =* 0.09, *F*_(1,70)_ *=* 0.561; *p =* 0.49]. Similarly, the levels used for the left- or right-ear stimuli did not correlate with rate discrimination in those ears [Left: *r =* 0.05, *F*_(1,71)_ *=* 0.17, *p =* 0.68, Right: *r =* 0.03, *F*_(1,71)_ *=* 0.077, *p =* 0.78]. As an additional check, we tested whether stimulus level expressed relative to T-level could account for ITD discrimination performance for the five listeners for whom accurate T-levels were obtained. The effect of worse ear remained significant when this measure was entered as a covariate [Left: *r =* 0.36, *F*_(1,48)_ *=* 6.52, *p =* 0.01, Right: *r =* 0.36, *F*_(1,48)_ *=* 7.14, *p =* 0.01], and this measure did not correlate with rate sensitivity [Left: *r =* 0.10, *F*_(1,48)_ *=* 0.46, *p =* 0.50, Right: *r =* 0.16, *F*_(1,48)_ *=* 1.21, *p =* 0.28]. Note that the levels used for each stimulus were loudness balanced for each listener. Hence, the absence of a correlation between those levels or dynamic ranges and either ITD or rate discrimination means that there is no evidence for any common factor underlying the across-electrode variation between these suprathreshold tasks and the current level needed to obtain a given loudness.

Together, these additional correlation analyses support the conclusion that worse-ear rate discrimination performance accounted for a significant proportion of the variance in ITD scores, independent of current level, and that this effect only occurred when the ITD score was compared to the rate discrimination performance from the matching electrode location.

### Comparison of Overall Performance in Rate and ITD Tasks

Performance was generally better overall for the rate discrimination task than that for the ITD task (circles often fall below squares in Fig. 1). This trend was analyzed by two separate repeated measures ANOVAs, with fixed factors of electrode position, rate, and task: ITD discrimination and either left- or right-ear rate discrimination. Both of these ANOVAs revealed a significant main effect of task [*F*_(1,6)_ = 20.15, *p* = 0.004 for left-ear rate versus ITD sensitivity, and *F*_(1,6)_ = 26.99, *p* = 0.002 for right-ear rate versus ITD]. It is important to note that because the differences between the standard and signal, 35 % for the rate task and 500 μs for the ITD task, were not equated in any way, this does not imply the presence of an additional source of limitation for the ITD task. Recall that the task design aimed to use a sufficiently large difference so that performance would be very good at 100 pps. A smaller rate difference, for instance, may have led to performance that was similar to, or even smaller than, that in the ITD task. However, one consequence of the better performance in the rate task is that it may have reduced the size of the between-task correlations, because it would have limited the number of conditions where performance in both tasks was above floor and below ceiling.

## DISCUSSION

### Comparison with Previous Studies

An extensive psychophysical literature has shown that sensitivity to differences in the temporal properties of electrical stimulation deteriorates markedly at rates above 300 pps, as compared to lower rates. This is true both for monaural (Shannon 1983; Tong and Clark 1985; Tong et al. 1982; Townshend et al. 1987; McDermott and McKay 1997; Zeng 2002; Kong et al. 2009; Carlyon et al. 2008, 2010b) and binaural processing (van Hoesel 2007; 2008; Carlyon et al. 2008; van Hoesel et al. 2009). The current study examined unilateral and bilateral rate sensitivity in the same CI listeners to disentangle whether unilateral and bilateral rate sensitivities were limited by similar or different sources.

The results presented here for unilateral rate discrimination are consistent with measures reported in previous studies. For most listeners, rate discrimination performance scores decreased monotonically with increasing rate, with a few exceptions where performance was somewhat lower at 100 pps than at 200 pps, as has been reported previously (Kong et al. 2009). Similarly, ITD discrimination was best at the lower rates and deteriorated gradually with increasing rate, as also shown in previous studies (Majdak et al. 2006; van Hoesel 2007; van Hoesel et al. 2009). In addition, for both tasks, performance did not differ significantly across electrodes when combined across listeners but could differ across electrodes in an idiosyncratic fashion for individual subjects. Similar findings have been reported elsewhere in the literature (for a recent review, see Kan and Litovsky 2014). It is worth noting that van Hoesel et al. (2009), who also showed no overall effect of electrode when data for apical, middle, and basal electrodes were entered into an ANOVA, did find slightly but significantly lower ITD thresholds for apical than for basal electrodes. In contrast, Best et al. 2011 and Laback et al. 2015 found consistently better performance for basal than for apical stimulation. For the current results, a repeated measures ANOVA showed that, even when data from the middle electrodes were removed from the analysis, there was no significant difference between performance at the apical and basal sites [*F*_(1,6)_ *=* 0.21, *p =* 0.66 for main effect of site of activation, ignoring the middle electrode pair]. There was a significant interaction between electrode and rate when the data from all electrodes was combined [*F*_(6,36)_ *=* 2.44; *p =* 0.044], but inspection of Figure 1H reveals that this was due to performance deteriorating *more* markedly with increasing rate at the apical electrode than at the mid or basal electrodes.

Two previous studies have compared performance in binaural and monaural tasks across listeners. One study measured ITD and rate discrimination thresholds in three bilaterally implanted users (van Hoesel 2007). To compare performance on the two tasks, at each rate, ITD thresholds were subtracted from the interpulse interval at that rate, and the resulting differences were expressed as percentages of the interpulse intervals. As noted in that study, the function relating this threshold to pulse rate was shallower than the function relating monaural rate discrimination thresholds to the standard rate, which led the author to suggest that monaural rate sensitivity is limited by factors that do not affect ITD perception (van Hoesel 2007). A subsequent study, however, questioned this subtraction-and-normalization method of transforming thresholds, on the basis that the proposed metric did not correspond to any calculation that was likely to be performed by the auditory system (Carlyon et al. 2008). In addition, both studies pointed out that performance in the ITD task may have been aided, at high rates, by increased reliance on the first pulse in each train and that this cue would not have been useful for the rate discrimination task (van Hoesel 2007; Carlyon et al. 2008). Perhaps more importantly, both studies found evidence that, for CI listeners, the high-rate deterioration in rate discrimination occurred even when a binaural cue was made available, such that listeners did not have to make explicit pitch judgments. They compared monaural (van Hoesel 2007) or diotic (Carlyon et al. 2008) rate discrimination with a condition in which one ear received a standard stimulus, and a signal, both containing different rates; the other ear received the standard rate stimulus. This resulted in a situation where the standard interval consisted of a diotic pulse train, and where, in the signal interval, the pulse rates differed between the two ears. Both studies found that this manipulation improved rate discrimination at low rates by allowing listeners to discriminate between a fused and a more diffuse binaural image, but that at rates where monaural and diotic rate discrimination broke down, the availability of this potential binaural cue did not help. This is consistent with the interpretation that the processing of fine timing differences at high repetition rates is limited by a factor that is common both to tasks that do and do not involve binaural processing.

### Possible Biological Basis of High-Rate Limitations

The present results are consistent with the idea that there is a common limitation restricting the processing of both ITD and rate cues at high overall pulse rates. An emerging literature on auditory physiology suggests that this limitation could occur at one or multiple stages along the auditory neuraxis, and some putative mechanisms are discussed below.

Evidence against one possible source of peripheral limitation, the auditory nerve, comes from a study by Carlyon and Deeks (2013), who compared evoked compound action potential recordings and rate discrimination in the same human listeners; they concluded that there was sufficient information in the compound neural response to support rate discrimination, even at high rates where actual performance was often at chance. Adding to the evidence against an auditory nerve limitation, a recent study on computational modeling of medial superior olive (MSO) units showed that although electric stimulation can severely distort the ability of auditory nerve fibers to follow the temporal fine structure of sound (Javel and Viemeister 2000), ITD sensitivity in model MSO units is comparable between acoustic and electric stimulation (Chung et al. 2015).

At a more central processing stage, the inferior colliculus, single-unit physiological recordings from anesthetized animals have shown that although many cells respond in a synchronized manner to pulse trains at low rates, these sustained responses become increasingly limited to onset-only responses at pulse rates higher than about 100 pps (Smith and Delgutte 2007; Hancock et al. 2012); sustained responses to somewhat higher rates have been found in the unanesthetized rabbit (Chung et al. 2014). Although these studies were primarily concerned with ITD processing, a neural response that was restricted to the first pulse in a stimulus would clearly be incapable of encoding pulse rate. Furthermore, Hancock et al. (2012) have argued that these onset-only responses may be due to the operation of low-voltage-activated potassium channels, which, being present in neurons at many brain stem sites, including the cochlear nucleus, are likely to influence the processing of monaural as well as of binaural stimuli. It is also known that the proportion of cells that do exhibit sustained responses at high rates depends somewhat on the duration of deafness, being greater in acutely deafened than in congenitally deaf cats (Hancock et al. 2012).

The finding that the history of auditory deprivation can affect the prevalence of sustained responses in the IC is relevant to the current findings if, as argued above, these sustained responses are important both for the coding of pulse rate and of ITD. This does not, however, necessarily imply that the effects reported here are mediated by experience-induced changes at the level of the IC; the common source of limitation could arise downstream to the inferior colliculus. Moreover, studies of auditory deprivation have compared responses between animals with different exposure histories, rather than across different places of excitation in the same animal. The current analyses partialed out between-subject effects, and although the onset and extent of neural damage may vary across different cochlear regions, we know of no studies that have investigated such region-specific deprivation. However, it is clear that a psychophysical study such as ours cannot identify the specific biological basis for the limitation in performance at high rates; the major contribution is to constrain the neural mechanisms to those involved both in the processing of monaural rate and fine interaural timing differences.

## SUMMARY

The results presented here compare worse-ear rate discrimination performance with ITD discrimination performance in bilaterally implanted CI listeners. Results corroborate previous findings that both monaural rate discrimination and ITD discrimination deteriorate with increasing pulse rate over the range 100–500 pps. No main effect of electrode position was observed for either task, and for the rate discrimination task, performance was, on average, similar for the two ears. However, performance did sometimes differ idiosyncratically for a given listener as a function of electrode location or (for rate discrimination) ear of presentation. By measuring performance in both tasks with the same set of listeners, the current results show that once the main effects of listener, rate, and electrode are removed, there is a significant correlation between rate discrimination in the worse ear and ITD discrimination. It is, therefore, to some extent possible to predict a listener’s ITD discrimination score for a given pair of electrodes stimulated at a given rate by taking into account the lower of the rate discrimination scores for the two electrodes in that pair. These results support the conclusion that the deterioration in both monaural and binaural temporal processing at high rates is at least partly due to a common mechanism. In contrast, the current level needed to reach a given loudness did not predict performance on either the ITD or rate discrimination tasks.
